# Supplementary material for: The interactome of intact mitochondria by cross-linking mass spectrometry provides evidence for coexisting respiratory supercomplexes
Source: Mol Cell Proteomics. 2017 Dec 8;17(2):216–32. doi: 10.1074/mcp.RA117.000470 (PMC5795388; doi:10.1074/mcp.RA117.000470)
Supplement: Supplemental Data [file supp_RA117.000470_133922_0_supp_23978_fzffwf.zip › spectra_annotation/mito_spectra_annotation/450-1-1-1-2-1.pdf]

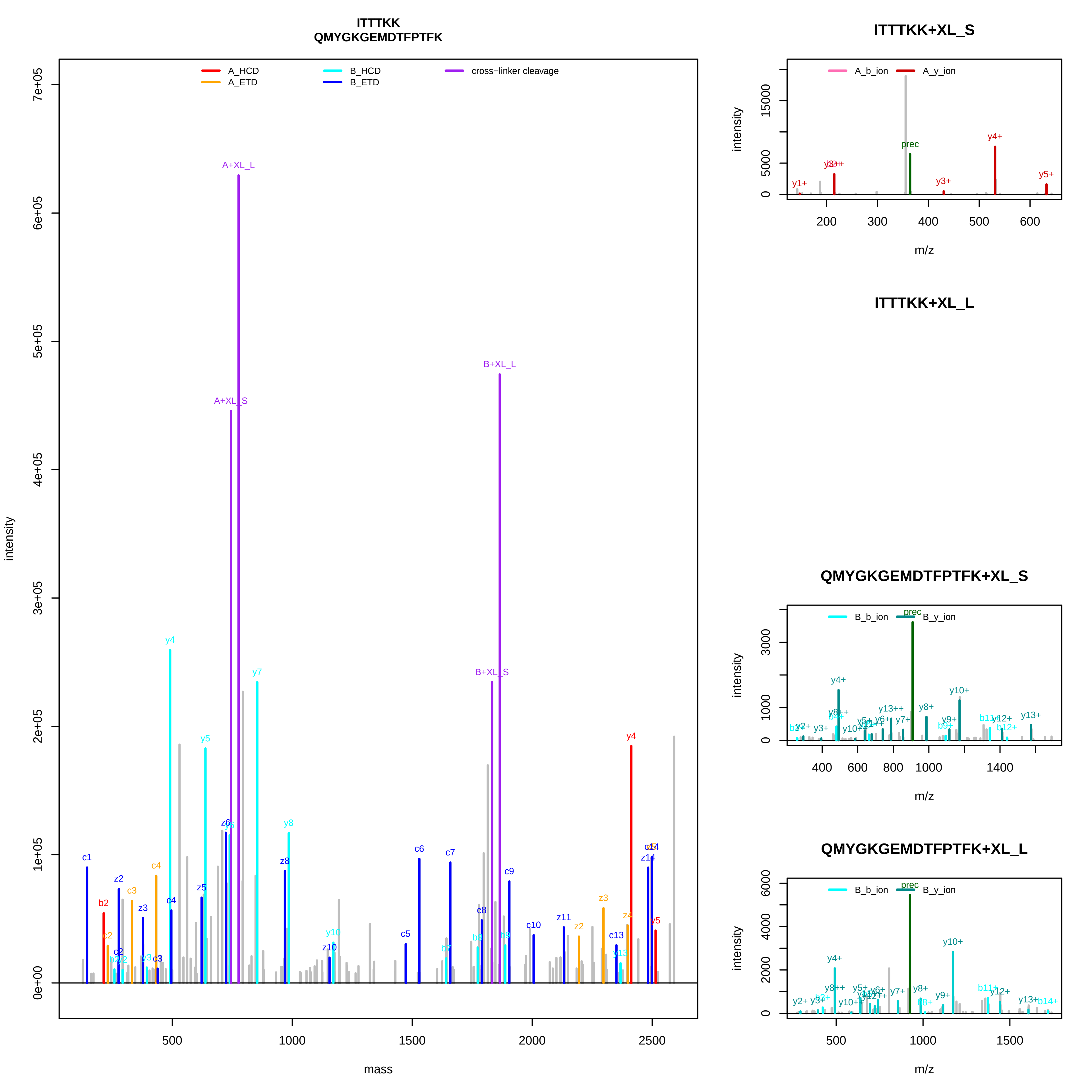
ITTTKK
QMYGKGEMDTFPTFK
A_HCD
A_ETD
B_HCD
B_ETD
cross−linker cleavage
intensity
mass
0e+00
1e+05
2e+05
3e+05
4e+05
5e+05
6e+05
7e+05
500
1000
1500
2000
2500
A+XL_L
A+XL_S
B+XL_L
B+XL_S
y4
y5
y7
y8
z6
c1
b2
c2
z2
c3
z3
c4
z5
z8
y10
z10
c5
c6
c7
c8
c9
c10
z11
z2
z3
z4
c13
y13
y4
y5
c14
z14
ITTTKK+XL_S
A_b_ion
A_y_ion
intensity
m/z
0
5000
15000
200
300
400
500
600
y1+
y3++
prec
y3+
y4+
y5+
ITTTKK+XL_L
QMYGKGEMDTFPTFK+XL_S
B_b_ion
B_y_ion
intensity
m/z
0
1000
3000
400
600
800
1000
1400
prec
y4+
y10+
y8+
y13++
y12+
y13+
QMYGKGEMDTFPTFK+XL_L
B_b_ion
B_y_ion
intensity
m/z
0
2000
4000
6000
500
1000
1500
prec
y4+
y10+
y8++
y7+
y8+
y9+
b11+
y12+
y13+
b14+
y2+
y3+
y10++
